# The addition of metformin to systemic anticancer therapy in advanced or metastatic cancers: a meta-analysis of randomized controlled trials

**DOI:** 10.7150/ijms.50338

**Published:** 2020-09-12

**Authors:** Hyeong Su Kim, Jung Han Kim, Hyun Joo Jang, Jin Lee

**Affiliations:** 1 Division of Hemato-Oncology, Department of Internal Medicine, Hallym University Medical Center, Hallym University College of Medicine, Seoul 07441, Republic of Korea.; 2 Division of Gastroenterology, Department of Internal Medicine, Dongtan Sacred-Heart Hospital, Hallym University Medical Center, Hallym University College of Medicine, Hwasung 18450, Gyeonggi-Do, Republic of Korea.

**Keywords:** metformin, cancer, randomized controlled study, prognosis, meta-analysis

## Abstract

Preclinical studies have demonstrated that metformin has anticancer properties and act in additive or synergistic way when combined with anticancer agents. We conducted this meta-analysis of randomized clinical trials to evaluate the effect of metformin added to systemic anticancer therapy in patients with advanced or metastatic cancer. A computerized systematic electronic search was performed using PubMed, PMC, EMBASE, Cochrane Library, and Web of Science databases (up to June 2020). From nine randomized clinical trials, 821 patients were included in the pooled analyses of odds ratios (ORs) with 95% confidence intervals (CIs) for overall response rate (ORR) and hazard ratios (HRs) with 95% CIs for progression-free survival (PFS) and overall survival (OS). The concomitant use of metformin with systemic anticancer therapy did not increase tumor response (the pooled OR of ORR = 1.23, 95% CI: 0.89-1.71, *p* = 0.21), compared with anticancer therapy alone. In terms of survival, metformin added to anticancer agents failed to prolong PFS (HR = 0.95, 95% CI: 0.75-1.21, *p* = 0.68) and OS (HR = 0.97, 95% CI: 0.80-1.16, *p* = 0.71). In conclusion, this meta-analysis of randomized clinical trials indicates that the addition of metformin to systemic anticancer therapy has no clinical benefits in patients with advanced or metastatic cancer.

## Introduction

Metformin is a biguanide that is commonly prescribed in the treatment of type 2 diabetes mellitus (DM). The mechanism of action by which metformin lowers blood glucose level is not fully understood, but it is known to decrease hepatic gluconeogenesis and increase insulin sensitivity 1. Recently, it has raised worldwide attention for its potential antitumor effects. Epidemiologic studies have reported that metformin use was associated with reduced risk of cancers among diabetic patients 2-6. In addition, experimental studies have demonstrated that metformin has antitumor effects *in vivo* and *in vitro*, which may repress proliferation of cancer cells and induce apoptosis, autophagy, and cell cycle arrest 7-11.

Metformin may act in additive or synergistic way when combined with anticancer therapy. Indeed, many preclinical studies have demonstrated the synergistic interaction of metformin and chemotherapeutic agents, such as gemcitabine 12, docetaxel 13, and platinum 14, or epidermal growth factor receptor-tyrosine kinase inhibitors (EGFR-TKIs) 15. Observational studies have also found the beneficial effects of metformin use for diabetic patients with advanced cancer 16-18. In a retrospective study of breast cancer (BC) patients treated with neoadjuvant chemotherapy, a high frequency of complete pathological responses was noted in diabetic patients treated with metformin, compared with diabetic patients prescribed with other anti-diabetic agents while receiving chemotherapy 16. Similarly, the analysis of data from the Surveillance, Epidemiology, and End Results registry data linked to medicare claims revealed that metformin significantly improved survival [hazard ratio (HR) = 0.80, 95% confidence interval (CI): 0.71-0.89] in metastatic non-small-cell lung cancer (NSCLC) patients with DM even after adjusting for demographics, diabetes severity and treatment, cancer characteristics, and oncologic treatment 17. Other retrospective study of patients with advanced pancreatic cancer (PC) undergoing chemotherapy, metformin therapy itself conferred better overall survival (OS) in comparison within DM patients (HR = 0.693, 95% CI: 0.492-0.977, *P* = 0.036) 18. In addition, single-arm phase II trial reported that metformin combined with 5-fluorouracil showed a modest but intriguing activity in patients with refractory metastatic colorectal cancer 19.

Based on these results, several randomized controlled trials have been conducted to investigate the effect of adding metformin to anticancer agents in patients with various types of cancer 20-30. Contrary to the observational studies, the most randomized trials failed to demonstrate a significant improvement of clinical outcomes in patients treated with metformin in combination with systemic anticancer treatment 20-27. However, the results were inconclusive because most studies were phase II trials with a small sample size. Therefore, we conducted this meta-analysis of randomized clinical trials to evaluate the effect of adding metformin to systemic anticancer therapy.

## Materials and methods

### Search strategy

This meta-analysis followed the Preferred Reporting Items for Systematic Reviews and Meta-Analyses (PRISMA) guidelines 31,32. A computerized systematic electronic search was performed using PubMed, PMC, EMBASE, Cochrane Library, and Web of Science databases (up to June 2020). The search included the following terms: “metformin” AND “carcinoma” or “cancer” or “neoplasm” or “malignancy” AND “randomized.” Besides, manual searching of references in identified studies and relevant reviews was performed to retrieve every potential article.

### Inclusion criteria

Eligible studies should meet the following inclusion criteria: (i) randomized clinical trials in human cancers; (ii) randomization of patients to systemic anticancer therapy with or without metformin; (iii) sufficient data for odds ratio (OR) with 95% confidence interval (CI) for overall response rate (ORR) and/or hazard ratio (HR) with 95% CI for progression-free survival (PFS) or OS; (iv) trials published only in peer-reviewed journals. There were no language or district restrictions.

### Exclusion criteria

We excluded: (i) non-randomized trials; (ii) cohorts with no control groups; (iii) trials that did not report essential outcomes (ORR, OS, or PFS); (iv) studies adding metformin to local anticancer treatments and, (v) abstracts not formally published in peer-reviewed journal.

### Data extraction

A pair of investigators (JH Jang and HS Kim) independently carried out the abstract screening, full text reviewing, and data extraction. Any disagreements were resolved by discussion, with input from the other investigator (JH Kim).

The following data were extracted from the included articles: first author, year of publication, trial phase, number of patients, primary endpoint, treatment setting and regimen, intervention in the control group, adverse events (AEs), ORR, and PFS and OS along with their 95% CI. If several estimations were conducted in one study, the most powerful result was selected (i.e., the multivariate regression would be given priority, and the univariate regression was superior to the unadjusted Kaplan-Meier analysis).

### Statistics

ORs, and HRs along with their 95% CIs used in the analyses were directly extracted from the original articles. If these statistical variables were not given explicitly in an article, they were calculated from available numerical data using methods reported by Parmar *et al.* 33.

The RevMan version 5.3 was used to combine the data. The heterogeneity across studies was estimated by using the I^2^ inconsistency test and chi-square-based Cochran's Q statistic test. The fixed-effect model based on Mantel-Haenszel method was selected when there was no substantial heterogeneity (*p* ≥ 0.1 or I^2^* ≤*  50%). When significant heterogeneity was observed (*p* < 0.1 and I^2^ >  50%), the random-effects model based on DerSimonian-Laird method was used. We also planned to perform additional subgroup analyses to identify the source of heterogeneity. Sensitivity analysis was conducted to assess the impact of each study on the pooled HR and heterogeneity by removing one study at a time.

Outcomes are shown as forest plots with diamonds representing the estimate of the pooled effect. The line of no impact is number one for binary outcomes, which depicts statistical significance if not crossed by the diamond 34. The pooled OR < 1.0 and HR < 1.0 implies higher rate and better survival, respectively, for the addition of metformin to anticancer therapy. The significance of the pooled HR and OR was determined by the *Z*-test, and the level of statistical significance was established as *p* < 0.05. Publication biases were evaluated graphically by the Begg's funnel plot and quantified by the Egger's test to assess funnel plot asymmetry 35,36.

### Quality of the included studies

The methodological quality of the randomized trials was scored by the Jadad five-item scale, assessing randomization, double blinding process, and withdrawals or dropouts 37. The final score ranged from 0 to 5, with low quality studies having a score ≤ 2 and high quality studies having a score of ≥ 3.

### Ethics

This study did not require the approval of the institutional ethics committee because it was a meta-analysis with systematic review of previously published articles.

## Results

### Results of search

**Figure 1:**
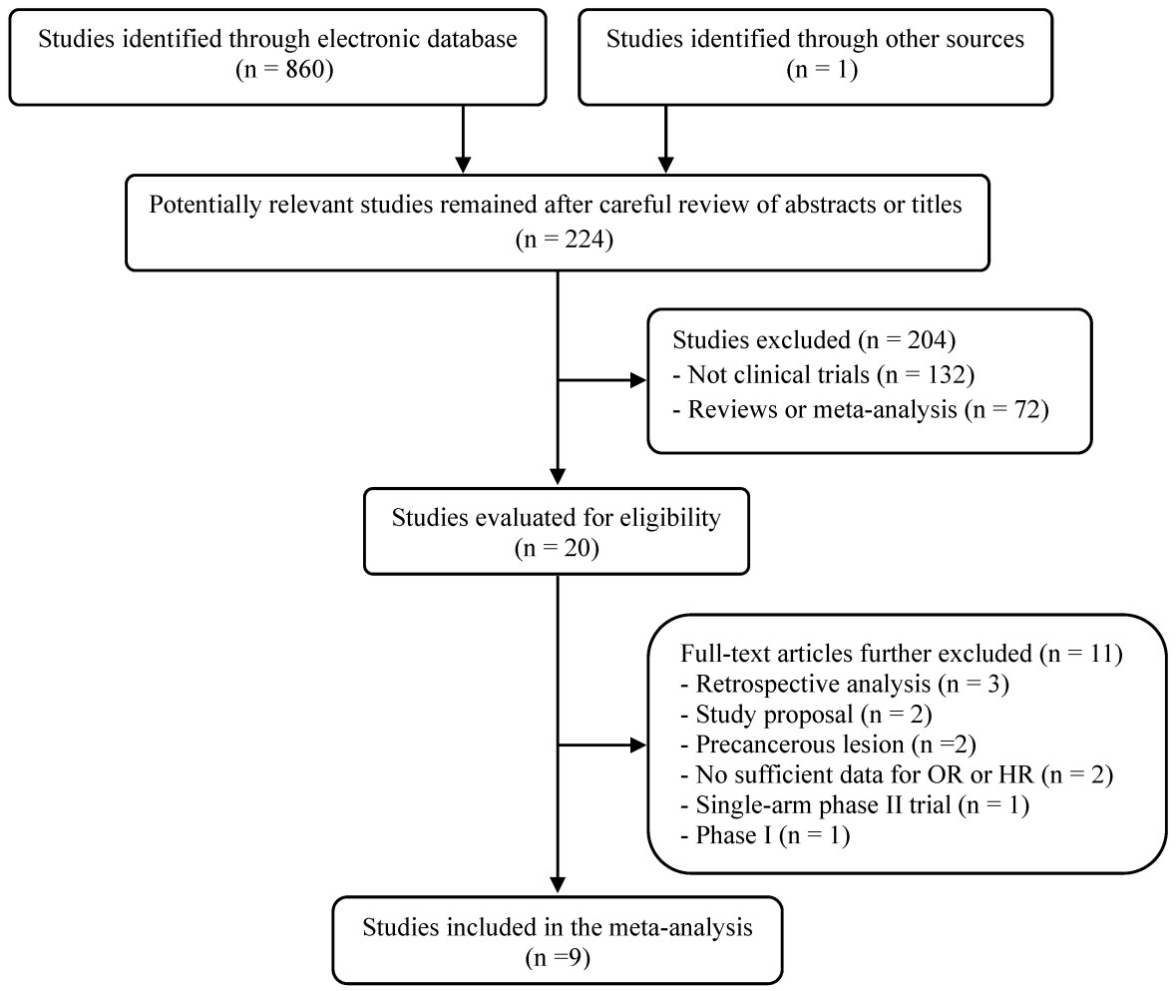
Flow diagram of search process.

The flow diagram of the search process is shown in **Figure 1.** A total of 224 potentially relevant articles were initially retrieved. Out of them, 204 articles were excluded after careful reviewing of the titles and abstracts. Of remaining 20 potentially eligible studies, 11 were further excluded by the inclusion or exclusion criteria. Eventually, nine randomized, clinical trials fulfilling the eligibility criteria were included in the meta-analysis 20-28.

### Characteristics of the included studies

**Table 1** summarizes the major characteristics and clinical outcomes of the nine included studies. All the studies were randomized phase II trials. The studies enrolled patients with advanced or metastatic PC 20,22, BC 23,25,26, and NSCLC 21,24,27,28. The dose of metformin used was from 500 mg/d to 2 g/d. Jadad score was more than 3 in all the studies, indicating a good quality of each study. From the nine studies, 821 patients were included in the meta-analysis.

### Effect of metformin addition on overall response rate

**Figure 2:**
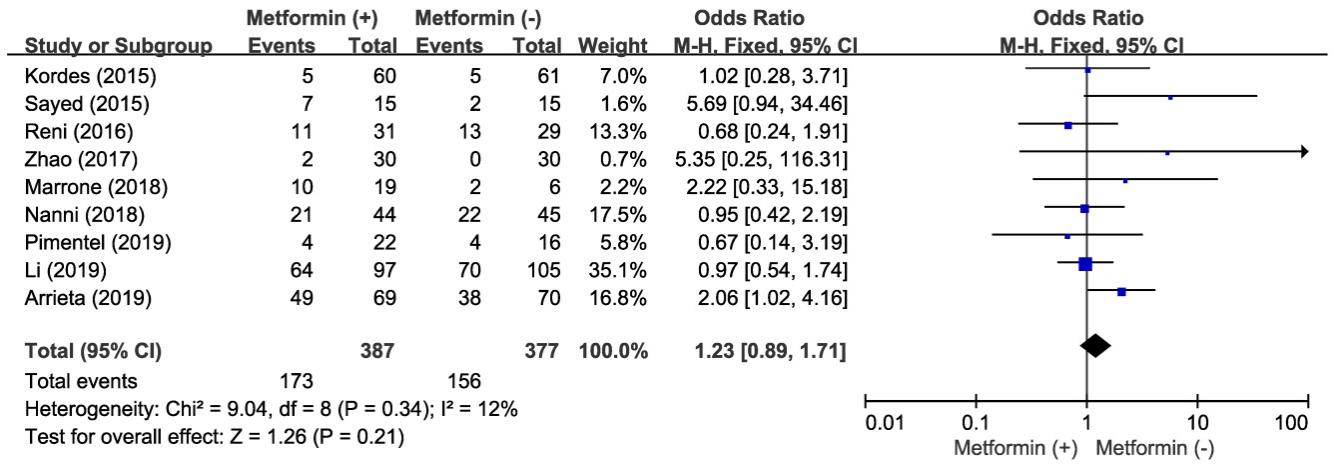
Forest plot for overall response rate.

There was no significant heterogeneity across the studies (χ^2^ = 9.04, *p* = 0.34, I^2^ = 12%) and the fixed-effect model was selected. The addition of metformin to anticancer agents did not increase ORR (OR = 1.23, 95% CI: 0.89-1.71, *p* = 0.21) (**Figure 2**).

### Effect of metformin addition on progression-free survival

**Figure 3:**
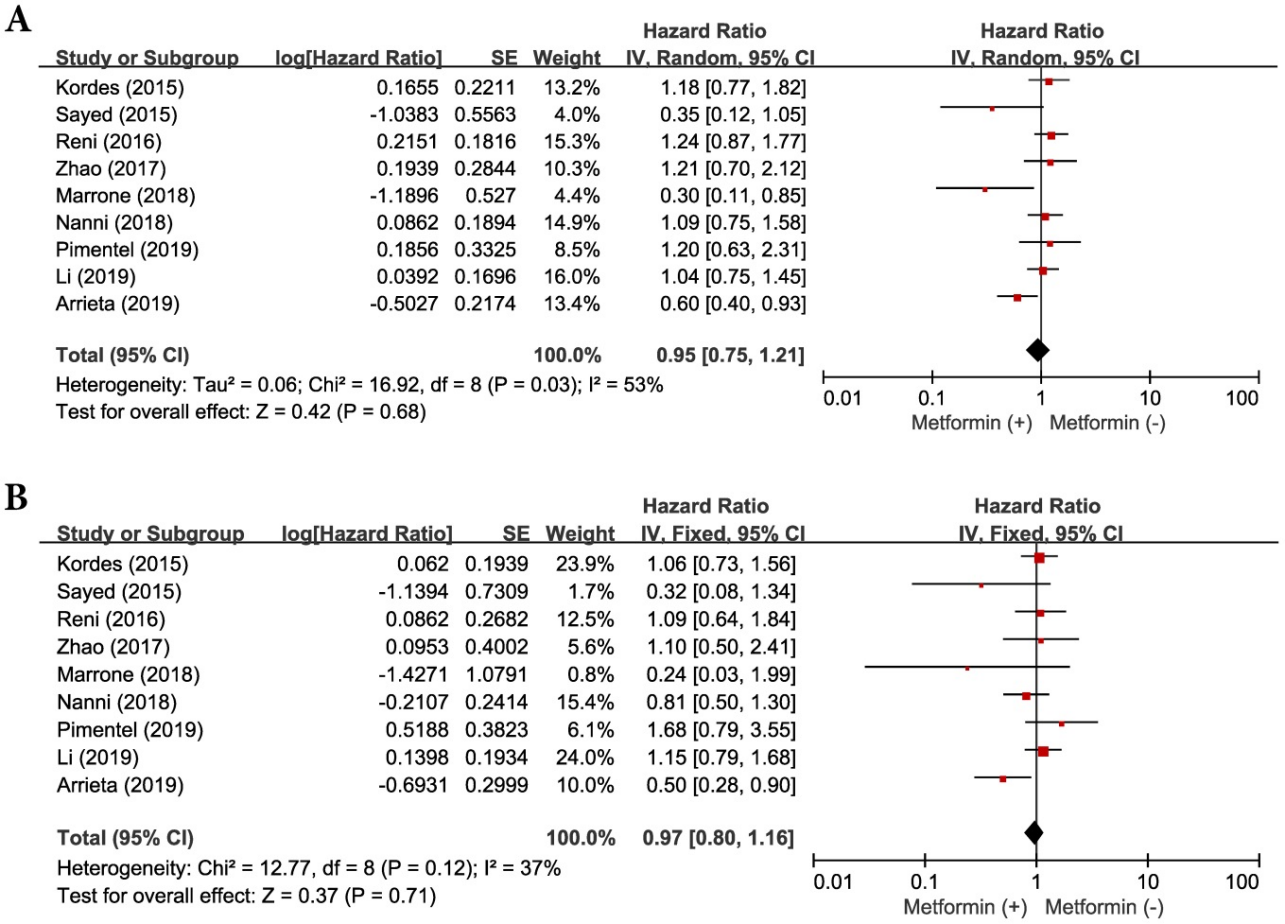
Forest plots for progression-free survival (**A**) and overall survival (**B**).

Because there was a significant heterogeneity across the studies (*χ*^2^ = 16.92, *p =* 0.03, I^2^ = 53%), the random-effects model was selected. The concomitant use of metformin and systemic anticancer therapy did not prolong significantly PFS (HR = 0.95, 95% CI: 0.75-1.21, *p* = 0.68) (**Figure 3A**), compared with anticancer therapy alone.

### Effect of metformin addition on overall survival

There was no significant heterogeneity among the studies (χ^2^ = 12.77, *p =* 0.12, I^2^ = 37) and the fixed-effect model was selected. Metformin added to anticancer therapy showed no significant impact on OS (HR = 0.97, 95% CI: 0.80-1.16, *p* = 0.71) (**Figure 3B**).

### Publication bias

**Figure 4:**
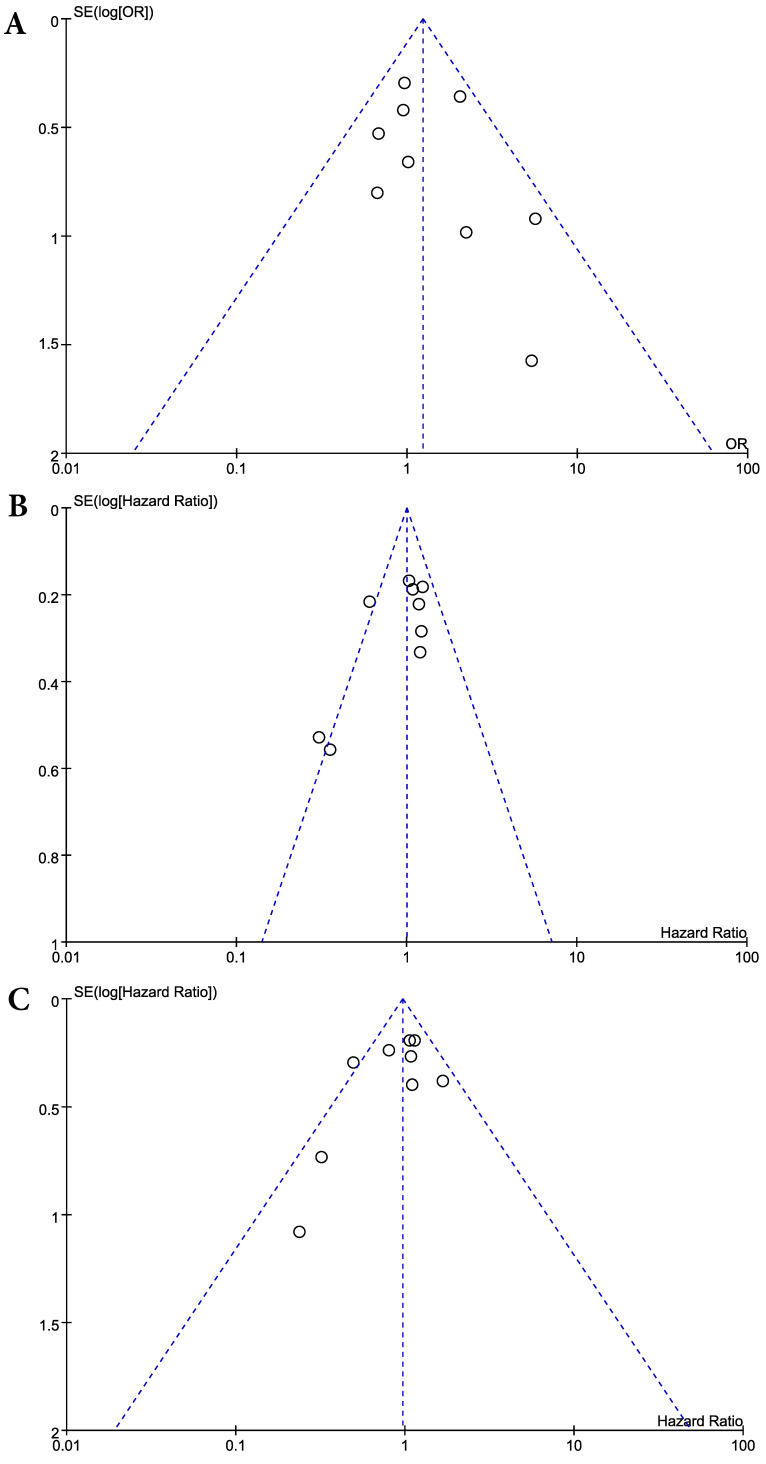
Funnel plots for publication bias: overall response rate (**A**), progression-free survival (**B**), and overall survival (**C**).

Visual inspection of the funnel plots for ORR, PFS, and OS showed symmetry, suggesting there was no substantial publication bias (**Figure 4**). Egger's tests also indicated the absence of significant publication biases (*p* = 0.375 for ORR, *p* = 0.095 for PFS, and *p* = 0.192 for OS).

## Discussion

Based on the preclinical and epidemiologic evidence of its anticancer effect 2-11, there have been growing interests in the effect of metformin among patients with cancer. Although both retrospective data and observational studies point to metformin having a potential role in cancer treatment 16-18, the anticancer effect of this drug has not been convincingly validated in prospective trials. This meta-analysis was conducted to assess the role of metformin in the fight against cancer. The results indicated that the addition of metformin to systemic anticancer therapy was not associated with improved clinical outcomes in patients with advanced or metastatic cancer.

Metformin alters cellular energy metabolism and is known to decrease hepatic glucose production in diabetes via adenosine monophosphate kinase (AMPK) dependent and independent mechanisms 1,38. The mechanisms of anticancer effects of metformin are controversial whether this activity is due to changes in the host metabolic environment or a result of direct action on cancer cells 39,40. One proposed mechanism involves its effect on decreasing insulin and insulin growth factor 1 (IGF-1) and lowering the levels of IGF-1 receptor and insulin receptor. In addition, metformin may have direct inhibitory effects by activating the AMPK protein, a serine/threonine kinase activated in adenosine monophosphate rich states and hypoxia 41. The AMPK in turn phosphorylates and inactivates proteins in the mTOR pathway, a regulatory pathway that inhibits cell proliferation, polarity, and division 42.

Consistent with experimental findings 7-11, epidemiologic and observational studies have reported that metformin was associated with reduction in the incidence of and mortality from various cancers 2-6,43. Based on these results, several randomized controlled trials have been conducted to investigate the effect of metformin added to anticancer therapy in patients with advanced or metastatic cancer 20-29. However, no studies indicated the addition of metformin to anticancer agents to be more effective, compared with anticancer therapy alone.

The first randomized controlled trial of metformin addition to standard treatment was conducted by Kordes *et al.* in patients with advanced PC 20. Patients were randomly assigned to receive gemcitabine and erlotinib with either metformin (up to 2g/d) (n = 61) or placebo (n = 60). However, there was no difference on OS between the two groups (median 7.6 in the placebo group vs. 6.8 months in the metformin group, *p* = 0.78). Reni *et al.* performed an open-label, randomized phase II trial in patients with metastatic PC 22. They randomly assigned 60 patients to receive cisplatin, eiprubicin, capecitabine and gemcitabine (PEXG) with (n = 31) or without metformin (2g/d) (n = 29). The trial was terminated early for futility after the preplanned interim analysis. Despite the differences in the study design between the two trials (e.g., disease stage, chemotherapeutic agents, blinding with use of placebo, interim analysis with early termination) 20,22, the results were consistent with no significant PFS and OS benefits in the metformin group. A couple of meta-analyses in PC suggested the beneficial effect of metformin on survival 44,45. However, they included not only randomized trials but also observational studies.

Several clinical studies have also suggested the antitumor effects of metformin in patients with BC. A retrospective study reported that patients treated with metformin during neoadjuvant chemotherapy achieved a higher pathologic complete response (pCR) rate than diabetic and non-diabetic patients not administered metformin 16. In the METTEN study assessing the efficacy of adding metformin to neoadjuvant chemotherapy plus trastuzumab in early HER2-positive BC, however, metformin addition failed to show statistically significant superiority in the rate of pCR (66% vs. 59%, *p* = 0.589) and breast-conserving surgery (79% vs. 59%, *p* = 0.089) 29. There are three randomized phase II trials of metformin added to systemic anticancer therapy in patients with metastatic BC 23,25,26. Zhao *et al.* randomly assigned 60 postmenopausal patients with pre-treated hormone receptor positive metastatic BC to receive aromatase inhibitor plus metformin (n = 30) or placebo (n = 30) 23. Nanni *et al.* investigated the efficacy of metformin plus chemotherapy (n = 57) versus chemotherapy alone (n = 65) in the first-line treatment of HER2-negative metastatic BC 25. Pimentel *et al.* conducted a randomized, placebo-controlled phase II trial to investigate the effect of metformin added to standard chemotherapy. Forty non-diabetic patients with metastatic BC were allocated to receive chemotherapy with either metformin (n = 22) or placebo (n = 18). These phase II trials with a small sample size failed to show any clinical benefits (ORR, PFS or OS) of metformin added to endocrine therapy or chemotherapy.

In patients with NSCLC, there are two randomized trials of metformin addition to chemotherapy. Sayed *et al.* investigated the effects of metformin on clinical outcome of non-diabetic patients with stage IV NSCLC 21. Thirty chemo-naïve patients were randomly assigned to receive gemcitabine and cisplatin with (n = 15) or without metformin (500 mg/d) (n = 15). The ORR and median survival time were better in the metformin group (46.7% vs. 13.3%, *p* = 0.109 and 12 months vs. 6.5 months, *p* = 0.119), but the difference was not statistically significant in this trial with a limited number of patients. Marrone *et al.* conducted a randomized phase II study of metformin plus paclitaxel/carboplatin/bevacizumab in patients with chemotherapy-naïve advanced or metastatic non-squamous NSCLC 24. Patients were randomly assigned (3:1) to receive chemotherapy with (n = 18) or without metformin (2 g/d) (n = 6). The study was stopped early due to slow accrual and changes in the standard first-line therapy of advanced NSCLC. Although PFS was longer in the metformin group (9.6 months vs. 9.6 months, log-rank *p* = 0.024), there was no significant differences in the secondary endpoints of ORR (56% vs. 35%, *p* = 0.11) and OS (15.9 months vs. 13.9 months, *p* = 0.186) between the two groups.

Recently, data has also shown the synergistic association between metformin and EGFR-TKIs 15,46,47. A retrospective study reported that concurrent use of metformin and an EGFR-TKI conferred superior outcomes over TKI alone in terms of PFS (19 vs. 8.0 months) and OS (32 vs. 23 months). The synergistic effects may have stemmed from metformin-mediated reversal of epithelial-mesenchymal transition and inhibition of interleukin-6 signaling 48. There are two randomized trials of metformin addition to EGFR-TKIs in patients with *EGFR*-mutant NSCLC. Li *et al.* conducted a randomized, double-blind phase II trial investigating the combining effects of metformin and gefitinib in patients with advanced *EGFR*-mutant NSCLC 27. Treatment-naïve patients (n = 224) were randomly assigned to receive gefitinib plus either metformin (n = 112) or placebo (n = 112). The median PFS (10.3 vs. 11.4 months, *p* = 0.808) and median OS (22.0 vs. 27.5 months, *p* = 0.457) were not significant different between the two groups. Arrieta *et al.* investigated the effects of metformin plus TKIs compared with TKI alone in patients with *EGFR*-mutated lung adenocarcinoma 28. Patients were randomly allocated to receive TKIs (erlotinib, afatinib, or gefitinib) plus metformin or EGFR-TKI alone. Patients allocated to receive an EGFR-TKI plus metformin showed higher ORR (71% vs. 54.3%, *p* = 0.042) than patients treated with an EGFR-TKI alone. The median PFS (13.1 vs. 9.9 months, HR = 0.60; 95% CI: 0.40-0.94, *p* = 0.03) and OS (31.7 vs. 17.5 months, HR = 0.5; 95% CI: 0.28-0.90, *p* = 0.02) were significantly longer in the EGFR-TKI plus metformin group compared with the EGFR-TKI monotherapy group. These findings suggest that the use of metformin as concomitant treatment of lung adenocarcinoma may be a valuable addition to improve clinical outcomes. Because this trial was neither blinded nor placebo-controlled, however, the results need to be interpreted with caution. There were also possible concerns regarding the remarkably inferior ORR and OS in the TKI alone group in comparison with historical data on the efficacy of TKIs in *EGFR*-mutant NSCLC.

This meta-analysis of the nine randomized clinical trials revealed that metformin in combination with systemic anticancer therapy failed to draw any clinical benefits in patients with advanced or metastatic cancer. Therapeutic intervention with metformin in cancer is an attractive option, as it is a well-tolerated oral medication with minimal side effects. The addition of metformin to anticancer agents was not associated with increased incidence of AEs in most studies. However, the combination of metformin and systemic anticancer therapy did not increase tumor response (the pooled OR of ORR = 1.23, 95% CI: 0.89-1.71, *p* = 0.21), compared with anticancer therapy alone. In agreement with the ORR, metformin added to anticancer agents failed to prolong PFS (HR = 0.95, 95% CI: 0.75-1.21, *p* = 0.68) and OS (HR = 0.97, 95% CI: 0.80-1.16, *p* = 0.71).

Several possibilities may explain the reasons why the addition of metformin to anticancer agents failed to show clinical benefits in patients with advanced or metastatic cancer. First, the impressive effect of metformin in reducing the incidence of and mortality from cancer might be associated with time-related biases (e.g., immortal time bias) in some observational studies 49. Second, the dose of metformin used in the clinical trials might be insufficient. The optimal dose of metformin to display anticancer effects is not known. The metformin in the included trials were administered at the same dosage as usually used in the treatment of diabetes. With regard to the proposed direct action of metformin on tumor cells, the drug concentration achieved in tumor tissue are crucial. Therefore, conventional anti-diabetes doses of metformin might fail to reach a sufficient concentration to exhibit antitumor effect. Third, as we known, cancer is not a homogeneous disease entity. Therefore, antitumor effects of metformin may significantly differ according to anatomical site and molecular type of cancers. The proposed indirect action mechanism of metformin postulates that its anticancer effects depend on the changes in the host metabolic environment, such as a reduction of insulin concentration and a resulting decrease in the activity of IR-PI3K-mTOR signaling pathway 3,50. However, not all cancers are responsive to insulin, and there might be types of cancer in which the reduced insulin concentration is insufficient to show anticancer effects. Another hypothesis is that anticancer effect of metformin might be limited in advanced or metastatic setting with a large tumor burden 51. Indeed, many observational or retrospective studies have reported that metformin was associated with improved outcomes when used in patients with non-metastatic disease 16,44,45,52. In addition, Coyle *et al.* reported the meta-analysis suggesting metformin could be a useful adjuvant agent, particularly in colorectal and prostate cancer 53.

This study has some inherent limitations that need to be discussed. First, this pooled analysis included a limited number of studies with a small sample size. Second, there were no phase III trials included in the meta-analysis. Third, patients had different types of tumors and received various therapeutic regimens. We could not perform subgroup analysis according to the primary site of cancers because of the limited number of trials. Fourth, there was a significant heterogeneity across the studies when combining HRs for PFS. It could not be completely interpreted although the random-effects model was selected.

In conclusion, this meta-analysis of randomized clinical controlled trials do not support clinical benefits of metformin added to systemic anticancer therapy in patients with advanced or metastatic cancer. However, further investigations including phase III trials are needed to resolve the issues (dose of metformin, treatment setting, particular cancer type, or immunomodulatory effect) on the addition of metformin to systemic anticancer therapy.

## Figures and Tables

**Table 1:** Nine randomized controlled trials of metformin addition to systemic anticancer therapy

First author (year) [ref.]	Cancer type	Phase	Setting	Treatment arm	No. of patients	Primary endpoint	ORR	AEs	mPFS (mo)	HR for PFS (95% CI)	mOS (mo)	HR for OS (95% CI)	Jadad score
Kordes (2015) 20	PC	II	1^st^	Gemcitabine/erlotinib + metformin (up to 2 g/d)	60	OS	8.3%	17 (28.3%)^*^	4.1	1.18 (0.77-1.82)	6.8	1.06 (0.73-1.56) *P* = 0.78	5
				Gemcitabine/erlotinib + placebo	61		8.3%	28 (46.7%)^*^	5.4		7.6		
Sayed (2015) 21	NSCLC	II	1^st^	Gemcitabine/cisplatin + metformin (500 mg/d)	15	ORR	46.7%	4 (26.7%)^†^	5.54	0.35 (0.12-1.05) *P* = 0.062	12	0.32 (0.08-1.34) *P* = 0.119	3
				Gemcitabine/cisplatin	15		13.3%	10 (66.7%)^†^	5		6.5		
Reni (2016) 22	PC	II	1st	PEXG + metformin (2 g/d)	31	PFS	35.5%	NA	4.9	1.24 (0.87-1.77) *P* = 0.036	6.83	1.09 (0.64-1.84) *P* = 0.13	3
				PEXG	29		45%	NA	6.1		10.4		
Zhao (2017) 23	H(+) BC	II	≥2^nd^	Aromatase inhibitor + metformin (1 g/d)	30	PFS	6.7%	5 (16.7%)^‡^	4.7	1.21 (0.70-2.12) *P* = 0.48	30.9	1.1 (0.50-2.41) *P* = 0.81	5
				Aromatase inhibitor + placebo	30		0%	3 (10%)^‡^	6.0		32.4		
Marrone (2018) 24	Non-Sq NSCLC	II	1^st^	Paclitaxel/carboplatin/bevacizumab + metformin (2 g/d)	19	PFS	56%	10 (56%)^±^	9.6	0.30 (0.11-0.85) *P* = 0.024	15.9	0.24 (0.03-1.99) *P* = 0.186	3
				Paclitaxel/carboplatin/bevacizumab	6		33%	2 (33%)^±^	6.7		13.9		
Nanni (2018) 25	HER2(-) BC	II	1^st^	Doxorubicin/cyclophosphamide + metformin (2 g/d)	57	PFS	48%	31 (54%)^±^	9.4	1.09 (0.75-1.58) *P* = 0.653	34.4	1.09 (0.75-1.58) *P* = 0.382	3
				Doxorubicin/cyclophosphamide	65		49%	47 (72%)^±^	9.9		26.8		
Pimentel (2019) 26	BC	II	≥1^st^	Chemotherapy + metformin (1.7 g/d)	22	PFS	18.2%	7 (31.8%)^≠^	5.4	1.2 (0.63-2.31) *P* = 0.58	20.2	1.68 (0.79-3.55) *P* = 0.18	3
				Chemotherapy + placebo	18		25%	10 (58.8%)^≠^	6.3		24.2		
Li (2019) 27	EGFR-mutant NSCLC	II	1^st^	Gefitinib + metformin (500 mg, 2g/d)	112	PFS	66%	26 (23.4%)^≠^	10.3	1.04 (0.75-1.45) *P* = 0.8087	22.0	1.15 (0.79-1.68) *P* = 0.4571	5
				Gefitinib + placebo	112		66.7%	21 (18.9%)^≠^	11.4		27.5		
Arrieta (2019) 28	EGFR-mutant lung ADC	II	≥1^st^	EGFR-TKI + metformin (1 g/d)	69	PFS	71%	NA	13.1	0.60 (0.40-0.94) P = 0.03	31.7	0.5 (0.28-0.90) P = 0.02	5
				EGFR-TKI	70		54.3%	NA	9.9		17.5		

ADC, adenocarcinoma; EGFR, epidermal growth factor receptor; TKI, tyrosine kinase inhibitor; NSCLC, non-small-cell lung cancer; non-Sq, non-squamous; PC, pancreatic cancer; H(+), hormone positive; Her2(-), PEXG, cisplatin, epirubicin, capecitabine, and gemcitabine; AEs, adverse events; ORR, overall response rate; mOS, median overall survival; mPFS, median progression-free survival; mo, months; HR, hazard ratio; CI, confidence interval; NA, not available. * Vomiting, †Nausea, ±Grade 3-4 neutropenia, ‡Arthralgia, ≠Grade 3-4 adverse events.
